# Proteomic analysis of the effects of caffeine in a neonatal rat model of hypoxic‐ischemic white matter damage

**DOI:** 10.1111/cns.13834

**Published:** 2022-04-08

**Authors:** Liu Yang, Xuefei Yu, Yajun Zhang, Na Liu, Danni Li, Xindong Xue, Jianhua Fu

**Affiliations:** ^1^ 85024 Department of Pediatrics Shengjing Hospital of China Medical University Shenyang China; ^2^ 540418 Department of Pediatrics The Second Hospital of Dalian Medical University Dalian China; ^3^ Department of Anesthesiology Dalian Municipal Maternal and Child Health Care Hospital Dalian China

**Keywords:** caffeine, hypoxic‐ischemic, proteomics, white matter damage

## Abstract

**Aim:**

White matter damage (WMD) is the main cause of cerebral palsy and cognitive impairment in premature infants. Although caffeine has been shown to possess neuroprotective effects in neonatal rats with hypoxic‐ischemic WMD, the mechanisms underlying these protective effects are unclear. Herein, proteins modulated by caffeine in neonatal rats with hypoxic‐ischemic WMD were evaluated.

**Methods:**

We identified differential proteins and performed functional enrichment analyses between the Sham, hypoxic‐ischemic WMD (HI), and HI+caffeine‐treated WMD (Caffeine) groups. Confirmed the changes and effect of proteins in animal models and determined cognitive impairment via water maze experiments.

**Results:**

In paraventricular tissue, 47 differential proteins were identified between the Sham, HI, and Caffeine groups. Functional enrichment analyses showed that these proteins were related to myelination and axon formation. In particular, the myelin basic protein (MBP), proteolipid protein, myelin‐associated glycoprotein precursor, and sirtiun 2 (SIRT2) levels were reduced in the hypoxic‐ischemic WMD group, and this effect could be prevented by caffeine. Caffeine alleviated the hypoxic‐ischemic WMD‐induced cognitive impairment and improved MBP, synaptophysin, and postsynaptic density protein 95 protein levels after hypoxic‐ischemic WMD by preventing the HI‐induced downregulation of SIRT2; these effects were subsequently attenuated by the SIRT2 inhibitor AK‐7.

**Conclusion:**

Caffeine may have clinical applications in the management of prophylactic hypoxic‐ischemic WMD; its effects may be mediated by proteins related to myelin development and synapse formation through SIRT2.

## Graphical abstract

We evaluated the caffeine‐regulated proteins in neonatal rats with hypoxia‐induced WMD and identified 47 differentially expressed proteins. Functional enrichment analysis showed that these proteins were related to myelin development and axon formation. We confirmed in animal models that caffeine increased the expression of myelin basic protein (MBP), synaptophysin (Syp), and postsynaptic density protein 95 (PSD‐95) after WMD by upregulating SIRT2 expression; these increases were attenuated by the SIRT2 inhibitor AK‐7. These findings support the clinical value of caffeine for preventing WMD and indicate that its effect is mediated by proteins related to myelin development and synapse formation.

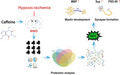

## 1 INTRODUCTION

White matter damage (WMD) is a common type of brain injury occurring in premature infants and is among the main causes of cerebral palsy and cognitive impairment.^1^, ^2^, ^3^ The main pathological features of brain WMD in preterm infants are brain atrophy, brain white matter area injury, and periventricular leukomalacia, which is characterized by oligodendrocyte maturation disorder and subsequent myelin sheath dysplasia.^4^, ^5^, ^6^ In addition, WMD also damages axon growth, synaptogenesis, and neurogenesis.^7^ Recent studies have suggested that the pathogenesis of WMD includes oxidative stress, inflammation, free radical damage, cytokine toxicity, and glutamate excitotoxic damage.^8^ Among these, oxidative stress and inflammation are the two main causes of WMD in premature infants.^9^ Although the etiology, pathogenesis, prevention, and treatment of WMD have been widely studied, effective neuroprotective strategies against WMD are still lacking.

Caffeine is a methylxanthine drug. Its early application in preterm infants can help prevent and treat apnea, can effectively reduce the incidence of bronchopulmonary dysplasia, and may exert long‐term beneficial effects on the nervous system.^10^ Caffeine can protect cells against oxidative stress and neuroinflammation, thereby reducing apoptosis and synaptic defects in developing neurons, reducing ventricular enlargement and white matter loss, and alleviating myelination disorders.^11^, ^12^ Additionally, caffeine exerts beneficial effects on neurodegenerative diseases, including Parkinson's and Alzheimer's disease.^13^, ^14^, ^15^ Although caffeine has been shown to exert neuroprotective effects on WMD in premature infants,^11^ the underlying neuroprotective mechanisms remain unclear.

Caffeine primarily acts as an antagonist of adenosine receptors at nontoxic doses/concentrations.^16^ In the brain, the activity of caffeine involves A1 and A2a adenosine receptors^17^ and indeed, “physiological” concentrations of caffeine only affect neuronal function through the action of these two adenosine receptors.^18^ Furthermore, the brain neuroprotection afforded by caffeine is fully accounted for by the antagonism of adenosine A2A receptors, as recently extensively reviewed.^19^ However, the molecular mechanisms engaged by the caffeine‐mediated antagonism of A2A receptors to induce this neuroprotection have not yet been identified.

Therefore, in this study, we examined the specific proteins and molecular mechanisms regulated by caffeine in a rat model of WMD caused by hypoxia‐ischemia.

## 2 METHODS

### 2.1 Experimental animals and study group establishment

All animal experiments were approved by the Animal Ethical Committee of China Medical University, Shenyang, China (approval no. 2017PS140K). Perinatal Sprague–Dawley rats were purchased from Liaoning Changsheng Biotechnology Co., Ltd. All rats were housed in a facility with a 12‐h light/dark cycle and were provided with free access to food and water.

There were approximately 10–16 newborn rats in each litter, and the birth weight was approximately 7.3 g (7.26 ± 0.325 g). All the rats were cross‐fostered between the dams. The animals were randomly divided into the following four groups: sham (Sham), hypoxic‐ischemic WMD (HI), HI+caffeine‐treated WMD (Caffeine), and caffeine with SIRT2 inhibitor (Caffeine+AK‐7) groups. In total, 187 animals were used in this experiment. From days 2–6 after birth, an intraperitoneal injection of 20 mg/kg/day caffeine citrate (Casey Pharmaceuticals) or an equal volume of normal saline was injected daily for 5 consecutive days. Rats in the caffeine+AK‐7 group were subjected to treatment with caffeine and the SIRT2 inhibitor AK‐7 (20 mg/kg/day, 5 days, intraperitoneal injection) from days 2–6 after birth.

### 2.2 Hypoxia‐ischemia‐induced brain WMD model in neonatal rats

Based on previously described methods,^7^, ^20^, ^21^ a rat model of neonatal brain WMD caused by hypoxia‐ischemia was established. Three‐day‐old male and female Sprague–Dawley neonates were anesthetized via subjection to inhalation of isoflurane and were fixed on an operating table in the supine position. The left common carotid artery was exposed under a dissecting microscope and was permanently ligated in the HI group using 2.0 sterile needle sutures. At both ends of the artery, the blood vessel was cut in the middle of the two ligature points, and the wound was sutured. The total operation time was 8–10 min. After completion of the operation, the rats regained consciousness and were housed with the mother to recover for 1 h; thereafter, they were placed in a hypoxic box in a constant temperature water bath (37°C). Mixed gas (8% O_2_ + 92% N_2_) was continuously provided as input for 2.5 h at a flow rate of 2 L/min, and the oxygen concentration was maintained at 8%. In the Sham group, the left common carotid artery was separated without ligation and hypoxia treatment.

The specimens were collected 14 and 21 days after model establishment, and 20 rats from each group were randomly selected at each time point for paraffin sectioning and western blot analysis; six randomly selected rats from each group were statistically analyzed. Additionally, all six rats in each group were subjected to Morris water maze (MWM) test after model establishment.

### 2.3 Sample preparation for proteomic analysis

Specimens were collected at 14 days after model establishment. After stripping the cortex, the brain tissues were isolated from the ligated side midbrain paraventricular areas on ice, which was rapidly flash‐frozen in liquid N_2_ and stored at −80°C. Frozen brain specimens were weighed and then ground in liquid nitrogen. Each sample was transferred to a 5‐ml centrifuge tube to which four volumes of lysis buffer (8 M urea, 1% Protease Inhibitor Cocktail) was added, and sonicated three times on ice using a high‐intensity ultrasonic processor (Scientz). The remaining debris was removed via centrifugation at 12,000 *g* at 4°C for 10 min. Finally, the supernatants were collected, and the protein concentration was determined using a BCA Kit according to the manufacturer's instructions (Beyotime Biotechnology). For digestion, the protein solution was subjected to reduction with 5 mM dithiothreitol for 30 min at 56°C and was alkylated with 11 mM iodoacetamide for 15 min at room temperature (24–26°C) in the dark. The protein sample was diluted by adding 100 mM tetraethylammonium bromide (TEAB) to a urea concentration of <2 M. Finally, trypsin was added (1:50 trypsin‐to‐protein mass ratio) for the first digestion overnight and at a 1:100 trypsin‐to‐protein mass ratio for the second 4‐h digestion.

### 2.4 Liquid chromatography tandem mass spectrometry (LC‐MS/MS) analysis

The tryptic peptides were dissolved in 0.1% formic acid in 2% acetonitrile (solvent A) and were directly loaded onto a reversed‐phase analytical column (15 cm length, 75 μm internal diameter) packed with 1.9 μm/120 Å ReproSil‐PurC18 resins (Dr. Maisch GmbH). The gradient was as follows: increase from 5% to 25% solvent B (0.1% formic acid in 90% acetonitrile) over 90 min, 25% to 35% in 22 min, increase to 80% in 4 min, and hold at 80% for the last 4 min. All steps were performed at a constant flow rate of 550 nl/min using an EASY‐nLC 1000 UPLC system (Thermo Fisher Scientific).

The peptides were subjected to nanospray ionization source followed by tandem mass spectrometry (MS/MS) using the Q Exactive Plus (Thermo Fisher Scientific) coupled online to an ultra‐performance liquid chromatography instrument. The electrospray voltage applied was 2.0 kV. The *m*/*z* scan range was 400–1500 for a full scan, and intact peptides were detected in the Orbitrap at a resolution of 60,000. Peptides were then selected for MS/MS by setting the normalized collision energy (NCE) to 20, and fragments were detected in the Orbitrap at a resolution of 15,000. A data‐dependent procedure was then performed that was alternated between one MS scan followed by 20 MS/MS scans with 15.0‐s dynamic exclusion. Automatic gain control was set at 5E4, and the fixed first mass was set to 100 *m*/*z*. The MS/MS data obtained were processed using the MaxQuant search engine (v1.6.15.0) and were explored against the Rattus norvegicus database (uniport database, 29940 sequence. Download 20201214) concatenated with a reverse decoy database. Extract the LFQ intensity in the database search result file, the row direction normalized by mean, and get the relative quantitative value of each sample.

### 2.5 Bioinformatic analysis

The R package clusterProfiler was used for Gene Ontology (GO) analysis, and Kyoto Encyclopedia of Genes and Genomes (KEGG) analyses were performed to predict the functions of differentially expressed peptides and their precursor proteins. Protein–protein interaction networks were mapped using the STRING database (https://string‐db.org/) and UniProt database (https://www.uniprot.org/). The isoelectric points and masses of peptides were determined using the tools available at https://www.expasy.org/ and https://smart.embl‐heidelberg.de/. Calculate the average value of the quantitative values of each sample in multiple replicates, and then calculate the ratio of the average values between the two samples. The relative quantitative values of each sample were taken as log2 transform (so that the data conforms to the normal distribution), and *p* value was calculated by the two‐sample two‐tailed T‐test method.

### 2.6 Immunohistochemistry

The coronal tissue sections used for the detection of paraventricular white matter ranged from 1.5 mm before bregma to 0.5 mm after bregma. The detection areas were mainly the corpus callosum (CC) and subventricular zone (SVZ) areas. At 14 and 21 days after model establishment, the rats were anesthetized by isoflurane inhalation and perfused transcardially with 0.9% saline in distilled water, followed by perfusion with 4% paraformaldehyde in 0.1 M phosphate‐buffered saline (PBS); their brains were postfixated in 4% formaldehyde in 0.01 M PBS for at least 48 h and then embedded in paraffin. After routine slicing of the brain tissues (3.0 μm), dehydrated slices were first dewaxed with xylene and were then hydrated using gradient ethanol solutions. They were heated for 30 min in citrate buffer (pH 6.0) for antigen retrieval, following which they were treated with 3% hydrogen peroxide for 20 min and blocked with goat serum for 30 min. The sections were incubated at 4°C overnight with the primary rabbit anti‐myelin basic protein (MBP) monoclonal antibodies (1:5000; cat. no. ab218011; Abcam) and mouse anti‐SITR2 monoclonal antibodies (1:250; cat. no. 66410‐1‐Ig; Proteintech). After rewarming, the samples were incubated with the secondary antibodies and streptavidin‐horseradish peroxidase for 20 min at 37°C, following which they were stained with 3,3'‐diaminobenzidine, restained, dehydrated, transparentized, and sealed. Images were obtained under a light microscope (Olympus Corporation) and were randomly selected and analyzed using the ImageJ software (NIH).

### 2.7 Immunofluorescence

After performing deparaffinization and heat‐mediated antigen retrieval, the tissue sections were blocked with goat serum for 30 min at 37°C, followed by incubation at 4°C overnight with the primary antibodies rabbit anti‐postsynaptic density protein 95 (PSD‐95) polyclonal antibodies (1:250; cat. no. 20665‐1‐AP; Proteintech) and rabbit anti‐synaptophysin polyclonal antibodies (1:100; cat. no. 17785‐1‐AP; Proteintech). After rewarming, the samples were incubated with a fluorescence‐labeled secondary antibody (1:200; Alexa Fluor 488; cat. no. ab150077; Abcam) for 4 h at room temperature. All sections were counterstained with 4′,6‐diamidino‐2‐phenylindole (DAPI). Immunofluorescence images were obtained using a confocal laser‐scanning microscope (C1; Nikon).

### 2.8 Luxol fast blue (LFB) staining

Brain slices were deparaffinized in xylene and were dehydrated in alcohol, immersed in 95% ethanol for 5 min at room temperature and then immersed in LFB solution (cat. no. G3245; Solarbio) overnight. The sections were immersed in 95% and 70% hydrochloric acid ethanol until gray and white matter could be distinguished.^22^

### 2.9 Western blotting

At 14 and 21 days after HI injury, rats were anesthetized with 3% isoflurane inhalation, the brains were harvested, and the brain tissues were isolated from the ligation side paraventricular areas on ice and were stored at −80°C. Samples were processed for western blotting, as per methods described previously.^23^ Brain tissue were lysed on ice in RIPA buffer containing phosphatase inhibitors (Sigma‐Aldrich). Protein concentrations were determined using a BCA kit (Sigma‐Aldrich). Protein samples (30 μg/sample) were separated by sodium dodecyl sulfate polyacrylamide gel electrophoresis and transferred to polyvinylidene fluoride membranes. The membranes were blocked with 5% fat‐free milk and then incubated with the following primary antibodies: rabbit anti‐MBP monoclonal antibodies (1:1000), mouse anti‐SITR2 monoclonal antibodies (1:2500), rabbit anti‐myelin‐associated glycoprotein (MAG) polyclonal antibodies (1:1000; cat. no. 14386‐1‐AP; Proteintech), rabbit anti‐myelin proteolipid protein (PLP) polyclonal antibodies (1:1000; cat. no. DF13282; Affinity Biosciences), rabbit anti‐PSD‐95 polyclonal antibodies (1:1000), and rabbit anti‐synaptophysin polyclonal antibodies (1:5000); rabbit anti‐β‐tubulin polyclonal antibodies (1:5000; cat. no. 10068‐1‐AP; Proteintech) was used as the loading control. After incubation with horseradish peroxidase‐conjugated goat anti‐rabbit antibodies (1:5000; cat. no. SA00001‐2; Proteintech) or anti‐mouse antibodies (1:5000; cat. no. SA00001‐1; Proteintech) and development using enhanced chemiluminescence reagents (Thermo Fisher Scientific), the intensities of all bands were analyzed using the ImageJ software and were normalized against β‐tubulin.

### 2.10 Morris water maze test

The Morris water maze (MWM) experiment was conducted from days 28 to 33 after model establishment.^24^ The test comprised the following: a circular pool (diameter: 160 cm; height: 60 cm), a black inner wall, a movable platform (diameter: 12 cm; height: 28.5 cm), a computer, a camera, and image‐ and data‐acquisition and processing systems. Different graphics were used to mark the pool wall at the midpoints of the four quadrants (Figure 2B). Before the commencement of the experiment, water was poured into the pool to a depth of 30 cm and the height of the platform was 1.5 cm below the water surface. The test included two phases, i.e., acquisition training and the probe trial. During the training period, the rats were subjected to training for 5 consecutive days, four times per day, to search for a platform for 120 s. Once a rat was found and remained on the platform for 5 s, the training was deemed complete. The time from entering the water to finding the platform was defined as the escape latency, and the system software automatically analyzed the swimming distance of the rat during this period. If the rat could not find the platform within 120 s, it was guided to rest on the platform for 20 s and the escape latency was recorded as 120 s. On day 6, the platform was removed and the probe trial was conducted, during which the rat was placed at the opposite quadrant and was allowed to swim for 120 s. The data were recorded using a video tracking system (Shanghai Mobile Datum Ltd.). All tests were performed by researchers who were blinded to the experimental groups. To evaluate the role of caffeine in cognitive impairment, the MWM test parameters, including the escape latency, time spent in the target quadrant, frequency of platform crossing (times), and moving velocity (moving distance/120) (cm/s), were evaluated.

### 2.11 Statistical analysis

All data are presented as the mean ± standard errors of means (SEMs). For normality assessment, the Kolmogorov–Smirnov test with Dallal–Wilkinson–Lillie correction for *p* values was used. Data related to latency escape or time were analyzed using two‐way repeated analysis of variance (ANOVA) followed by Bonferroni's post hoc test. All other results among three or more experimental groups were analyzed using one‐way ANOVA followed by Bonferroni's post‐hoc test if the data were normally distributed. If the data were not normally distributed, the Kruskal‐Wallis test was performed as a nonparametric test. GraphPad Prism software (v.8.01; GraphPad Software) was used for the analyses, and results with *p* < 0.05 were considered statistically significant.

## 3 RESULTS

### 3.1 Caffeine administration resulted in differential proteins involved in hypoxic‐ischemic WMD in neonatal rats

**FIGURE 1:**
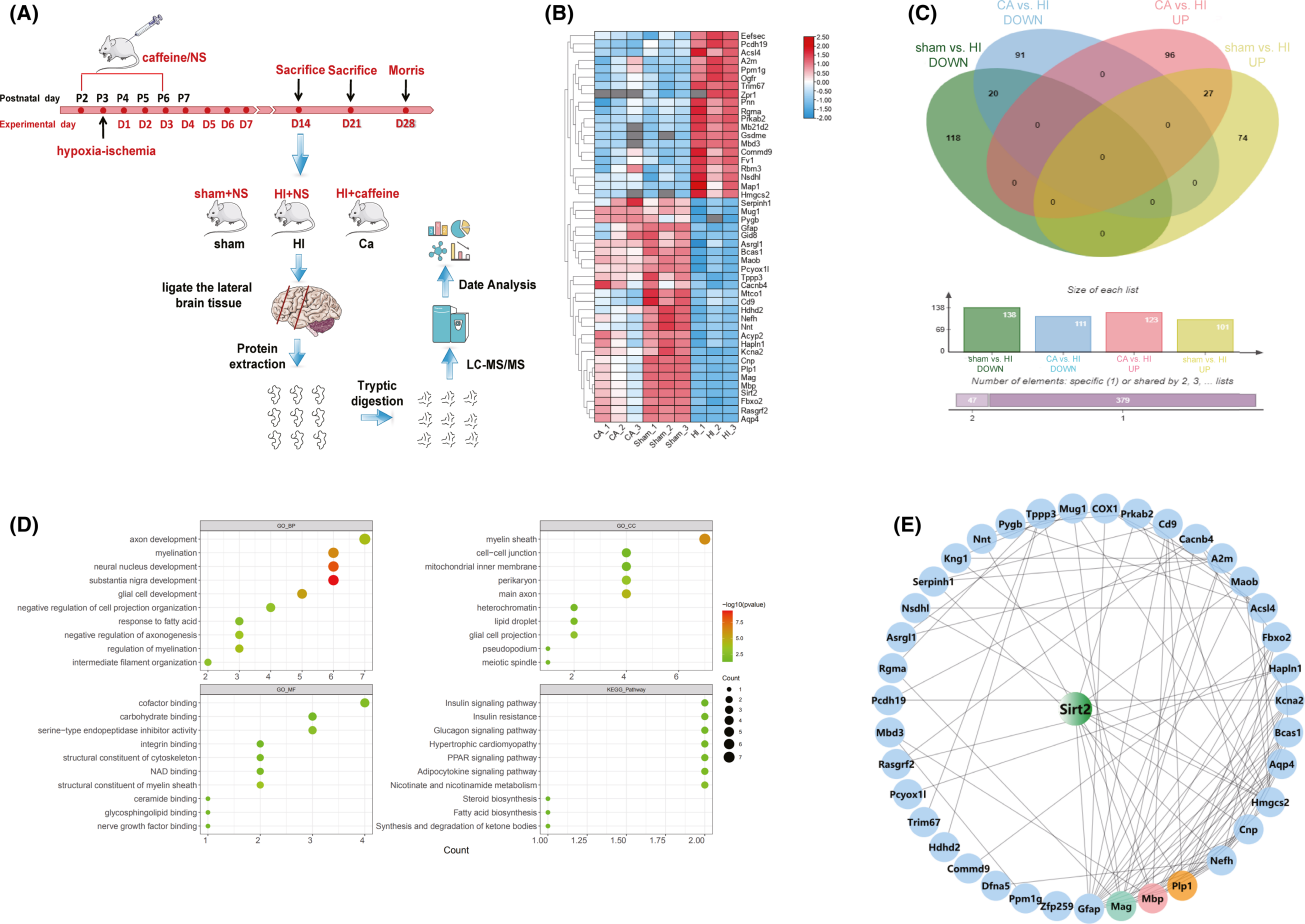
Differential protein identification and bioinformatics analysis of caffeine‐treated neonatal rats with hypoxic‐ischemic white matter damage. (A) Schematic overview of the proteomic analysis. LC‐MS/MS, liquid chromatography‐tandem mass spectrometry. (B) Heatmap illustrating protein abundance values derived from the brains of rats in the Sham, HI, and Caffeine groups (three rats per group). (C) Venn diagram depicting differentially expressed proteins. (D) Gene Ontology (GO) and Kyoto Encyclopedia of Genes and Genomes (KEGG) analysis. (E) Protein–protein interaction (PPI) networks. CA, Caffeine group; HI, HI group; Sham, Sham group

At 14 days after hypoxic‐ischemic model establishment, proteins were extracted from the brain tissues of three rats each from the Sham, HI, and Caffeine groups, and analyzed directly using LC‐MS/MS (Figure 1A,B). In total, 47 differential proteins (Sham vs. HI, *p* < 0.05 and fold change >1.2; HI vs. Caffeine, fold change >1.2) were identified (Figure 1C). Compared with those in the Sham group, the levels of 27 proteins were found to be upregulated in the HI group and downregulated in the Caffeine group, whereas the levels of 20 proteins were downregulated in the HI group and upregulated in the Caffeine group. We ranked these proteins according to their *P* values, and the proteins showing the greatest upregulation and downregulated are listed in Table 1.

**TABLE 1:** Top 10 upregulated and downregulated proteins in Sham group and Caffeine group compared with the HI group

Expression change	Protein description	Gene name	Protein accession	Coverage (%)	Sham/HI log_2_FC	CA/HI log_2_FC
UP	Myelin basic protein	Mbp	P02688	53.8	1.351	0.573
	Myelin proteolipid protein	Plp1	P60203	23.8	1.230	0.491
	Myelin‐associated glycoprotein	Mag	P07722	24.9	1.171	0.379
	2′,3′‐cyclic‐nucleotide 3′‐phosphodiesterase	Cnp	P13233	64.5	0.896	0.396
	Neurofilament heavy polypeptide	Nefh	F1LRZ7	21.5	0.745	0.270
	Murinoglobulin‐1	Mug1	Q03626	11.3	0.723	0.877
	Breast carcinoma‐amplified sequence 1 homolog	Bcas1	A0A0G2K079	54.5	0.706	0.429
	NAD‐dependent protein deacetylase	Sirt2	A0A0G2JWM2	53.6	0.650	0.287
	Acylphosphatase	Acyp2	D4A1G1	42.7	0.597	0.308
	F‐box only protein 2	Fbxo2	G3V774	35.8	0.589	0.381
DOWN	T‐kininogen 1	Map1	P01048	37	−2.620	−1.703
	Alpha‐2‐macroglobulin	A2m	P06238	6.9	−0.877	−0.364
	Tripartite motif‐containing 67	Trim67	D3ZTX1	15.7	−0.784	−0.454
	Protocadherin 19	Pcdh19	D3ZG18	4.4	−0.762	−1.059
	RNA binding motif protein 3	Rbm3	G3V6P6	51.9	−0.726	−0.325
	Hydroxymethylglutaryl‐CoA synthase	Hmgcs2	P22791	14.2	−0.680	−0.431
	COMM domain containing 9	Commd9	Q3MIE7	36.9	−0.638	−0.447
	Similar to zinc finger protein (predicted)	Zpr1	D3ZWN1	8.1	−0.606	−0.535
	5′‐AMP‐activated protein kinase subunit beta‐2	Prkab2	Q9QZH4	21.4	−0.565	−0.530
	Protein phosphatase 1G	Ppm1g	A0A0H2UHT5	28.6	−0.541	−0.319

Note

Protein description and protein accession are listed according to the SWISS‐PROT database; coverage (%), sequence coverage (%).

Abbreviations: CA, Caffeine group; FC, fold change; HI, HI group; Sham, Sham group.

### 3.2 Bioinformatics analysis showed that caffeine protected against hypoxic‐ischemic WMD in neonatal rats

To preliminarily explore the functions of the differential proteins in neonatal rats with hypoxic‐ischemic WMD, we used the R package clusterProfiler to analyze GO and KEGG pathway enrichment of the 47 overlapping expressed genes of differential proteins. In total, 125 GO biological processes, 16 GO cellular components, 27 GO molecular functions, and 11 KEGG pathways were enriched. In particular, we focused on detecting enrichment for myelin‐related pathways, including myelination, regulation of myelination, myelin sheath formation, and structural constituent of myelin sheath, and synaptic‐related pathways, including axon development, main axon formation, long‐term synaptic potentiation, and response to axon injury. We ranked the results according to the *P* values, and the top 10 enriched GO terms and KEGG pathways are shown (Figure 1D).

To identify important proteins mediating the protective effects of caffeine against brain WMD in neonatal rats, we used the STRING and UniProt databases to further evaluate the functions of differential proteins. We generated a protein–protein interaction network (Figure 1E) focusing on the interaction effects between MBP, PLP1, MAG, and SIRT2, which are related to axon formation and myelination.

### 3.3 Caffeine attenuated cognitive impairment related to hypoxic‐ischemic WMD in neonatal rats

**FIGURE 2:**
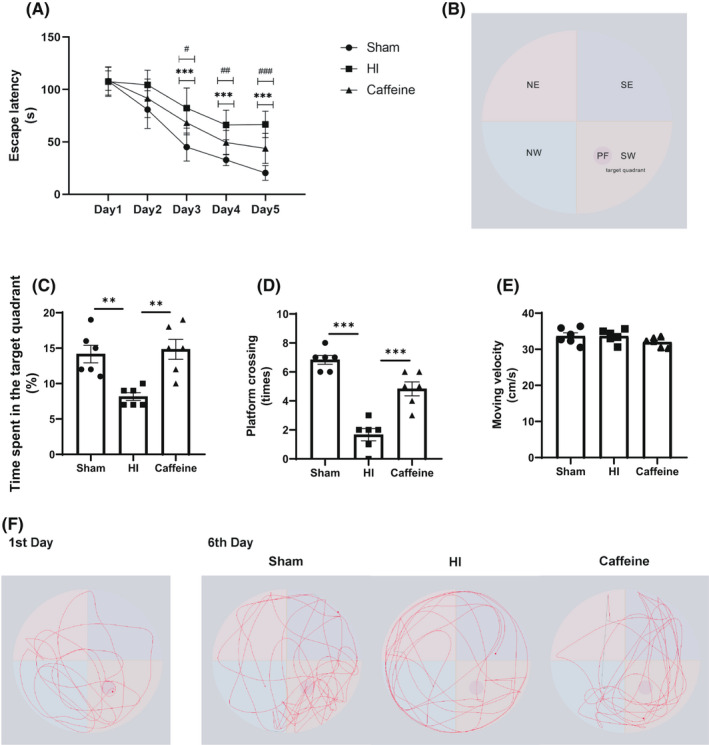
Caffeine attenuated cognitive impairment related to hypoxic‐ischemic WMD in neonatal rats. (A) Escape latency (s) of rats in the training trials of the hidden platform task. (B) Schematic representation of the water maze experiment zone. NE: northeast; SE: southeast; NW: northwest; SW: southwest; PF: platform. (C) Proportion of time spent (%) in the target quadrant in the probe trial. (D) Frequency of platform crossing (times) in the probe trial. (E) Moving velocity (cm/s) in the probe trial. (F) Trajectory graph of representative pathways on the first and last training days of hidden platform task for each group. Data are presented as means ± SEMs. Statistical analyses included two‐way (Latency escape) and one‐way ANOVA, followed by Tukey's test. */#*p* < 0.05, **/##*p* < 0.01, ***/###*p* < 0.001. #: HI vs. Caffeine group; *: Sham vs. HI group. Sham group (*n* = 6); HI group (*n* = 6); and Caffeine group (*n* = 6)

To verify the effects of caffeine on cognitive impairment associated with hypoxic‐ischemic WMD in neonatal rats, we performed a water maze experiment at 28 days after hypoxic‐ischemic model establishment. In the MWM experiment, the escape latency in the HI group on days 3–5 was found to be significantly longer than that in the Sham group (*p* all <0.001); this increase was attenuated by caffeine (Figure 2A). In spatial probe trials, rats in the HI group showed a lower proportion of exercise time spent in the target quadrant (*p* < 0.01) and demonstrated significant reductions in the number of times they crossed the platform (*p* < 0.001) compared with those in the Sham group. However, caffeine could attenuate the alterations in time spent in the target quadrant and platform crossing caused by HI (*p* < 0.01, *p* < 0.001) (Figure 2C,D,F). There were no significant differences in moving velocity within 120s among the three groups (Figure 2E). This indicated that the above‐mentioned differences could not be explained by differences in athletic ability among the groups. These results indicated that caffeine reduced the cognitive dysfunction of neonatal rats with brain WMD caused by hypoxia‐ischemia.

### 3.4 Caffeine prevented myelin development disorders related to hypoxic‐ischemic WMD in neonatal rats

**FIGURE 3:**
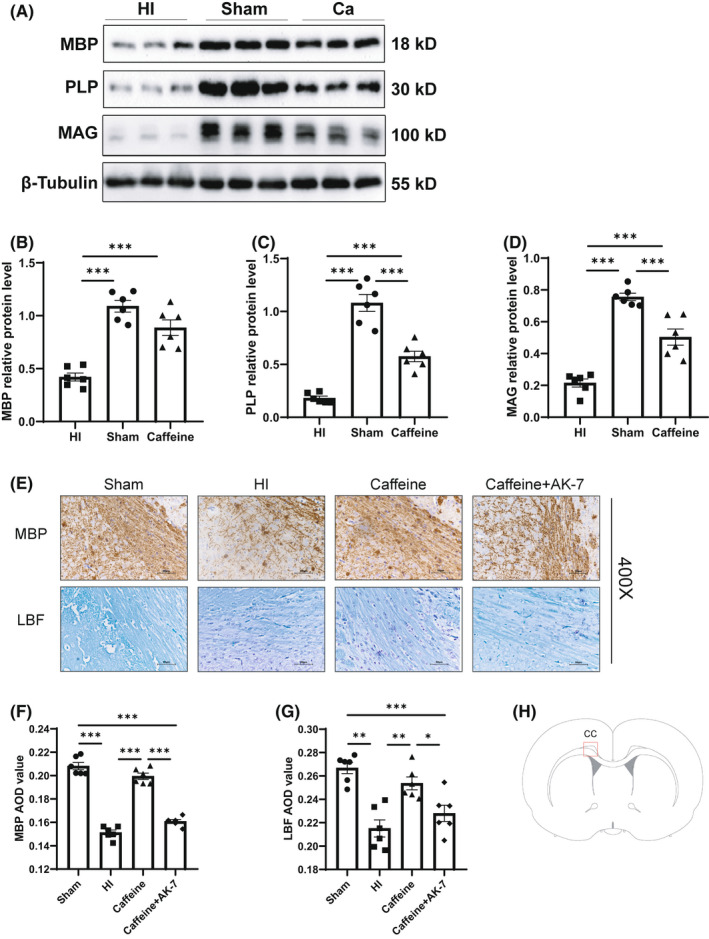
Caffeine improved myelin development after hypoxic‐ischemic WMD in neonatal rats. (A) Western blot detection of MBP, PLP, and MAG. Relative protein levels of (B) MBP, (C) PLP, and (D) MAG. (E) Immunohistochemical staining of MBP and LFB staining in the corpus callosum (CC) area of the ligated side cerebral hemisphere at 14 days after hypoxic‐ischemia (MBP scale bar = 50 μm; LBF scale bar = 50 μm). Average optical density (AOD) values of (F) MBP and (G) LBF staining. (H) Diagrammatic representation of the brain region analyzed for the level of MBP and LBF staining. Data are presented as means ± SEMs. Statistical analyses involved one‐way ANOVA, followed by Tukey's test. *p < 0.05, **p < 0.01, ***p < 0.001. Sham group (*n* = 6); HI group (*n* = 6); Caffeine group (*n* = 6); and Caffeine+AK‐7 group (*n* = 6). Ca, Caffeine group; Ca+AK‐7, Caffeine+AK‐7 group

Next, we explored the effects of caffeine on myelin sheath development in neonatal rats with hypoxic‐ischemic WMD by further evaluating differential proteins related to myelination, such as MBP, PLP, and MAG. As determined by western blotting, the protein levels of MBP, PLP, and MAG in the ligated cerebral hemisphere at 14 days after hypoxia‐ischemia in the HI group were significantly lower than those in the Sham group (*p* all <0.001), and caffeine was found to prevent the alterations in these proteins caused by HI (*p* all <0.001) (Figure 3A–D).

Myelination was examined via MBP immunohistochemical staining and LFB staining. At 14 days after hypoxia‐ischemia, the average optical density (AOD) values of MBP and LFB staining in the CC (Figure 3H) area of the ligated side cerebral hemisphere in the HI group were lower than those in the Sham group (*p* < 0.001, *p* < 0.01) (Figure 3F,G), and the arrangement of the nerve fibers was disordered and sparse (Figure 3E). The AODs for MBP and LFB staining in the Caffeine group were higher than those in the HI group (*p* < 0.001, *p* < 0.01) (Figure 3F,G). The nerve fibers were arranged in an orderly and dense manner, and the myelin sheath was partially remodeled (Figure 3E). These data indicated that caffeine alleviated myelination disorders after hypoxia‐ischemia‐induced WMD.

### 3.5 Caffeine prevented reductions in synapse proteins induced by hypoxic‐ischemic WMD in neonatal rats

**FIGURE 4:**
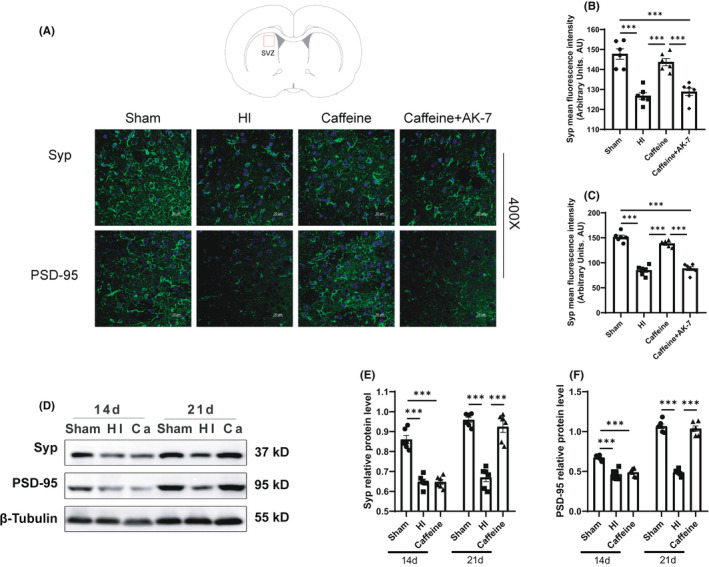
Caffeine improves synapse proteins after hypoxic‐ischemic WMD in neonatal rats. (A) Immunofluorescence staining of synaptophysin (Syp) and PSD‐95 (scale bar = 50 μm). (B) Mean fluorescence intensity of Syp. (C) Mean fluorescence intensity of PSD‐95. (D) Western blot detection of Syp and PSD‐95. (E) Relative protein levels of Syp. (F) Relative protein levels of PSD‐95. Data are presented as means ± SEMs. Statistical analyses included two‐way (Relative protein levels of Syp and PSD‐95 at different times) and one‐way ANOVA, followed by Tukey's test. *p < 0.05, **p < 0.01, ***p < 0.001. Sham group (*n* = 6); HI group (*n* = 6); Caffeine group (*n* = 6); and Caffeine+AK‐7 group (*n* = 6). Ca, Caffeine group; Ca+AK‐7, Caffeine+AK‐7 group

We then performed immunofluorescence and western blotting experiments to determine levels of the presynaptic protein synaptophysin (Syp) and postsynaptic protein PSD‐95. Western blotting results showed that the protein levels of Syp and PSD‐95 decreased at 14 and 21 days after hypoxia‐ischemia (*p* all <0.001), whereas caffeine prevented the HI‐induced reduction of Syp and PSD‐95 protein levels at 21 days after hypoxia‐ischemia (*p* all <0.001) (Figure 4D–F), with no significant difference between the caffeine and HI groups at 14 days after hypoxia‐ischemia. Immunofluorescence analysis showed that compared with the Sham group, the mean fluorescence intensity of Syp and PSD‐95 in the SVZ area of the ligated side cerebral hemisphere in the HI group were reduced at 21 days after hypoxia‐ischemia (*p* all <0.001), and caffeine prevented the alterations caused by HI (*p* all <0.001) (Figure 4A–C). These results suggested that caffeine improved the synapses proteins after hypoxic‐ischemic WMD in neonatal rats.

### 3.6 SIRT2 promoted the beneficial effects of caffeine on the prevention of hypoxic‐ischemic WMD in neonatal rats

**FIGURE 5:**
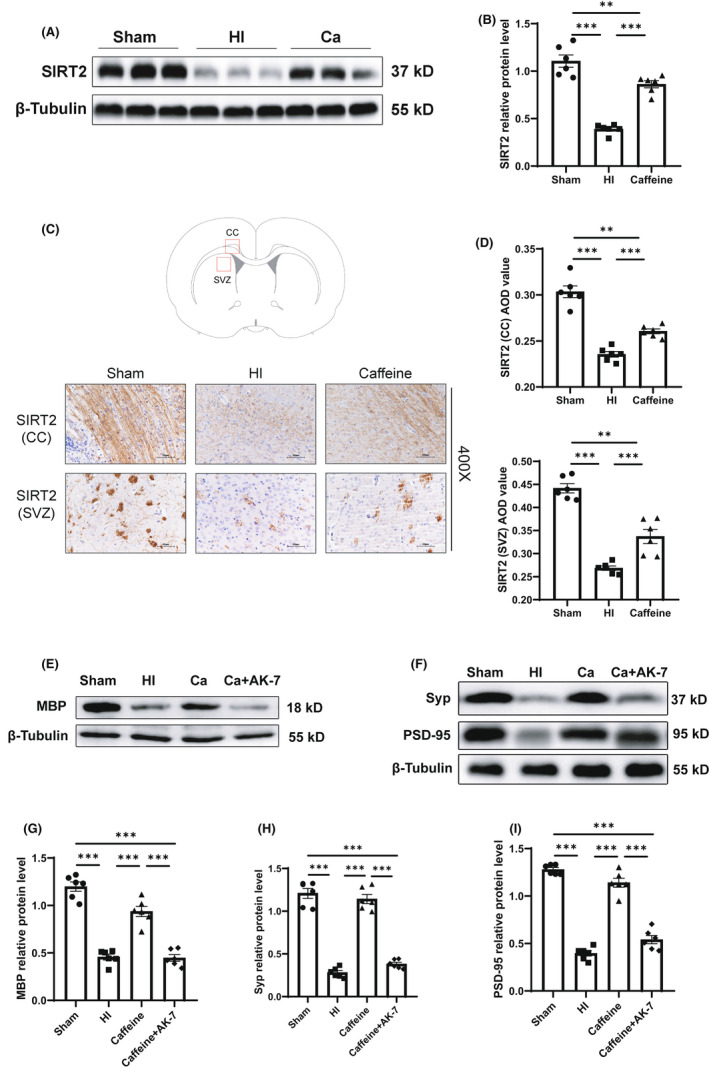
SIRT2 promoted the beneficial effects of caffeine on preventing hypoxic‐ischemic WMD in neonatal rats. (A) Western blot detection of SIRT2. (B) Relative protein level of SIRT2. (C) Immunohistochemical staining of SIRT2 in the corpus callosum (CC) and subventricular zone (SVZ). (D) Average optical density (AOD) value of SIRT2 in CC and SVZ. (E) Western blot detection of MBP. (F) Western blot detection of synaptophysin (Syp) and PSD‐95. Relative protein level of (G) MBP, (H) Syp, and (I) PSD‐95. Data are presented as means ± SEMs. Statistical analyses included one‐way ANOVA, followed by Tukey's test. *p < 0.05, **p < 0.01, ***p < 0.001. Sham group (*n* = 6); HI group (*n* = 6); Caffeine group (*n* = 6); and Caffeine+AK‐7 group (*n* = 6). Ca, Caffeine group; Ca+AK‐7, Caffeine+AK‐7 group

SIRT2 was one of the differential proteins identified in our proteomic analysis. We performed western blotting and immunohistochemistry to verify the changes in SIRT2 protein levels following treatment with caffeine to prevent the alterations caused by HI. Western blotting results showed that at 14 days after hypoxia‐ischemia, SIRT2 protein levels were lower in the HI than that in the Sham group (*p* < 0.001), and caffeine prevented the HI‐induced reductions in SIRT2 levels (*p* < 0.001) (Figure 5A,B). Consistent with the results of western blotting, AOD values of SIRT2 in the CC and SVZ area of the ligated side cerebral hemisphere in the HI group were found to be significantly lower than those in the Sham group at 14 days after hypoxia‐ischemia (*p* all <0.001), whereas these alterations caused by HI could be prevented by caffeine (*p* all <0.001) (Figure 5C,D).

We further explored the roles of SITR2 in the beneficial effects of prophylactic caffeine treatment in hypoxic‐ischemic WMD. Rats were subjected to treatment with caffeine and the SIRT2 inhibitor AK‐7 to evaluate changes in myelination and synapse proteins in WMD caused by hypoxia‐ischemia. Western blotting results showed that at 14 days after hypoxia‐ischemia, the protein level of MBP in the Caffeine+AK‐7 group was lower than that in the Caffeine group (*p* < 0.001) (Figure 5E,G). Furthermore, inhibition of SIRT2 level reversed the beneficial effects of caffeine. Consistent with the western blotting results, immunohistochemical analysis showed that MBP and LFB AOD values in the Caffeine+AK‐7 group were lower than those in the Caffeine group at 14 days after hypoxia‐ischemia (*p* < 0.001, *p* < 0.05) (Figure 3E–G). Western blotting results also showed that at 21 days after hypoxia‐ischemia, the protein levels of Syp and PSD‐95 in the Caffeine+AK‐7 group were lower than those in the Caffeine group (*p* all <0.001) (Figure 5F,H,I). The immunofluorescence results showed that the mean fluorescence intensities of Syp and PSD‐95 in the caffeine+AK‐7 group were also lower than those in the Caffeine group at 21 days after hypoxia‐ischemia (*p* all <0.001) (Figure 4A–C).

These results indicated that SIRT2 levels decreased in brain tissues after hypoxia‐ischemic WMD and that caffeine can help alleviate myelination disorders and improve synapse proteins by preventing HI‐induced reductions in SIRT2.

## 4 DISCUSSION

Hypoxic‐ischemic brain WMD in premature infants can lead to severe neurological dysfunctions, including movement disorders, cognitive and behavioral disorders, mental retardation, and visual and auditory disorders, with convulsions and epilepsy occurring in certain cases.^23^, ^25^, ^26^, ^27^, ^28^, ^29^ WMD in preterm infants involves the occurrence of early oligodendrocyte damage and subsequent myelination disorders.^30^, ^31^, ^32^ Hypoxia‐ischemia leads to the development of oligodendrocyte differentiation disorders, a decrease in mature oligodendrocytes, insufficient myelin synthesis, and myelination disorders.^33^ PLP, MAG, and MBP are three important proteins involved in myelination in the central nervous system. MAG is highly sensitive to ischemia and is only expressed on axons away from the oligodendrocyte body, whereas MBP and PLP are expressed throughout the myelin sheath.^34^ Reductions in PLP, MAG, and MBP levels have been observed in neurodegenerative diseases, such as Parkinson's and Alzheimer's disease, and in convulsions caused by brain WMD.^34^, ^35^, ^36^

Early studies have shown that caffeine exerts a neuroprotective effect on ischemic WMD.^11^, ^12^ Indeed, myelination is enhanced and ventriculomegaly is reduced in hypoxia‐exposed neonatal pups treated with caffeine from P3 to P12. These observations support that caffeine administration during early postnatal development may have applications in the prevention of periventricular white matter injury.^11^ Previous findings have also described the impact of caffeine on leukomalacia following perinatal ischemia and provided the first direct evidence that the targets of nontoxic doses of caffeine‐adenosine receptors affected oligodendrocyte maturation.^37^ Recent findings have shown that caffeine promotes oligodendrocyte differentiation and maturation by preventing hypoxia‐induced calcium overload.^38^ While confirming the neuroprotective effects of caffeine on premature encephalopathy, many studies have also confirmed its safety in the immature brain. An experiment on the effects of high‐dose caffeine exposure (25 mg/kg caffeine base loading dose; 20 mg/kg daily maintenance dose) on the development of brain white matter in immature sheep also showed that there was no effect of daily high‐dose caffeine on brain weight, oligodendrocyte density, myelination, axonal integrity, microgliosis, astrogliosis, apoptosis, or neuronal density.^39^ A recent study on the long‐term effects of caffeine treatment on the nervous system of premature rabbits model also shows that caffeine treatment had no significant effect on behavior between preterm infants and full‐term prepubescent individuals. Although the neuronal density of preterm infants is low, it is not affected by caffeine treatment. At the same time, there was no difference in synaptic density, astrocytes, and myelin sheath between the groups treated with or without caffeine.^40^ Our study found that continuous use of caffeine before and after brain injury could alleviate long‐term behavioral disorders caused by HI and played a neuroprotective role, which is consistent with the results of earlier studies. At the same time, we identified the specific proteins regulated by caffeine in hypoxic‐ischemic WMD, including MBP, PLP, and MAG. Regulation of the expression levels of myelination‐associated proteins is important for preserving the structural integrity and functionality of oligodendrocytes. Caffeine regulated the expression levels of myelination‐related proteins and attenuated the reduction in oligodendrocyte maturation caused by hypoxic‐ischemic WMD. In this study, the levels of myelination‐associated proteins (PLP, MAG, and MBP) were found to be decreased in hypoxic‐ischemic WMD, and caffeine alleviated this decrease.

Simple myelination disorder development cannot fully explain the diversity of long‐term sequelae in preterm infants with WMD. Studies have suggested that synaptic damage is an important pathological basis for this diversity.^41^ Synapse formation and remodeling are vital for brain development in premature infants.^42^ Researchers have reported synaptic damage in animal models of hypoxic‐ischemic encephalopathy,^43^ resulting in abnormal limb movement and cognitive memory, among other issues.^44^ There are numerous studies showing that caffeine controls synaptic dysfunction associated with neuropsychiatric disorders.^45^, ^46^, ^47^ A recent study identified the main targets of nontoxic doses of caffeine as a key controller of synaptic stability^48^ and showed that the exposure to caffeine during brain development affects susceptibility to brain dysfunction later in life.^49^ In addition, caffeine can also affect hippocampal synaptic plasticity through A1 and A2A receptors.^50^ Also, it has been shown that caffeine has profound effects on synaptic markers in younger age in rodents^51^ and prevents memory dysfunction later in life after challenges applied in early life.^52^ Caffeine treatment also prevents the sleep deprivation‐induced downregulation of synaptophysin and synapsin I proteins with no change in PSD‐95 protein in hippocampus.^53^ Prenatal caffeine intake increases the number of NeuN‐stained nuclei in the fetal brains of rats, and the maturation of telencephalon may be accelerated. At the same time, prenatal caffeine intake temporarily affects synaptic proteins and changes fetal brain development.^54^ We also found that although caffeine did not significantly enhance the protein levels of the presynaptic protein Syp or PSD‐95 at 14 days after hypoxia‐ischemia, the levels of both Syp and PSD‐95 were significantly increased at 21 days in the Caffeine group, suggesting that the protective effects of caffeine were related to increases in the expression of long‐term synapse proteins after WMD. However, the mechanisms through which caffeine upregulates myelin‐associated proteins and synaptic proteins remain unclear.

In our proteomics analysis, in addition to identifying the myelination‐related protein regulated by caffeine in hypoxic‐ischemic WMD, it is more important to identify the protein interacting with myelination‐related protein–sirtuin 2 (SIRT2). As a nicotinamide adenine dinucleotide‐dependent deacetylase, SIRT2 is mainly expressed in myelinating glia of the central nervous system (CNS) and plays an important role in neurological diseases.^55^, ^56^, ^57^, ^58^ Pathological features, such as neuroinflammation, synaptic dysfunction, metabolic abnormalities, and oxidative stress, are affected by SIRT2 expression.^55^ Early studies have shown a counterbalancing role of SIRT2 against a facilitatory effect of tubulin acetylation on oligodendroglial differentiation. Selective SIRT2 availability to oligodendroglia may have important implications for myelinogenesis, myelin‐axon interaction, and brain aging.^59^ Meanwhile, proteolipid protein is required for the transport of sirtuin 2 into CNS myelin.^60^ In recent years, research has proved that SIRT2 expression enhances OL differentiation and arborization in vitro,^61^ and plays a critical role in OL differentiation by promoting both arborization and downstream expression of myelin‐specific genes.^62^ In addition, SIRT2 also affects synaptic plasticity.

SIRT2 can act as a deacetylase for α‐amino‐3‐hydroxy‐5‐methyl‐4‐isoxazole propionic acid (AMPA), which interacts with the GluA1 subunit of AMPA receptor (AMPAR) and regulates AMPAR transport and protein homeostasis, thereby affecting learning and memory ability.^63^ Although SIRT2 is widely thought to accelerate the development of neuropathology,^55^ recent studies have shown that middle‐aged mice lacking SIRT2 exhibit axon degeneration and motor dysfunction.^64^ These contradictory findings indicate that SIRT2 level is necessary for normal axon formation; however, this protein functions differently after neurological injury. SIRT2 may have different effects on various brain injuries owing to the activation of diverse signaling pathways. Therefore, SIRT2 may function as a metabolic coordinator through deacetylation of substrate in disease. Our study found that SIRT2 was decreased in hypoxic‐ischemic induced WMD, and that caffeine could prevent the HI‐induced reductions of SIRT2. In the presence of the SIRT2 inhibitor AK‐7, caffeine failed to enhance the expression of myelinating proteins and synaptic proteins after hypoxia‐ischemia. We speculate that the beneficial effects of caffeine on myelinating and synaptic proteins may be achieved by preventing the reduction in SIRT2 induced by HI. Although we did not include a control group in which AK‐7 was applied alone in the HI group, many previous studies have revealed the safety of AK‐7. In order to determine the dose‐effect relationship of AK‐7, Chopra et al. first conducted tests using 10 and 20 mg/kg AK‐7 tests in male C57BL/6 mice and found that these doses had no obvious side effects on Huntington's disease model mice.^65^ In addition, a study on the inhibition of SIRT2 by AK‐7 in rats with traumatic brain injury showed that AK‐7 alone had no adverse effect in the control group, whereas AK‐7 after brain injury could aggravate neuroinflammation and blood‐brain barrier damage.^66^ We speculate that SIRT2 may be necessary for damage repair, but that its function may differ before then. Therefore, the exact roles and mechanisms of SIRT2 in biological processes should be further studied in order to make rational use of SIRT2 as a potential therapeutic target.

## 5 CONCLUSION

Our results indicated that caffeine may interfere with oligodendrocyte maturation and with synaptic function, conferring protection to neonatal brain tissue against damage caused by hypoxia and ischemia. However, a major finding was the alteration of SIRT2, a major metabolic coordinator. These findings led to obvious conclusion that metabolic rebalancing may be another candidate mechanism to explain the neuroprotective effects of caffeine, in accordance with the well‐established ergogenic effects of caffeine in sports physiology. However, we did not test the effects of AK‐7 in control rats or of AK‐7 alone in HI rats, and our study had some other limitations. Based on our findings, we hypothesize observed effects were just the summation of the two independent effects caused by caffeine and AK‐7. Therefore, further studies are needed to fully elucidate the molecular mechanisms involved. Based on the results obtained herein, we suggest that caffeine may be considered an effective drug for preventing WMD in premature infants; further studies on the molecular mechanisms and biological functions of caffeine may provide new insights into targets for promoting the use of caffeine as a neuroprotective agent.

## CONFLICT OF INTEREST

The authors have no conflicts of interest to declare.

## AUTHOR CONTRIBUTIONS

Jianhua Fu and Xindong Xue made substantial contributions to conception and design of the experiment. Liu Yang, Xuefei Yu, Yajun Zhang, Na Liu, and Danni Li contributed to the acquisition, analysis, and interpretation of data. All authors approved the final version of the manuscript for publication.

## CONSENT TO PARTICIPATE

All animal data reported in this study were obtained following ARRIVE guidelines. The animal study was reviewed and approved by the Chinese Council on Animal Research. The current study was approved by the Animal Ethics Committee of Shengjing Hospital of China Medical University [Reference number: 2017PS140K].

## Data Availability

The data that support the findings of this study are available from the corresponding author upon reasonable request.
